# A New Lignin-Based Polyurethane Film for Wood: Decay, Artificial Weathering, Physical and Morphological Characterization

**DOI:** 10.3390/molecules30244793

**Published:** 2025-12-16

**Authors:** Swati Tamantini, Sara Bergamasco, Miha Humar, Marko Petrič, Manuela Romagnoli

**Affiliations:** 1 Department for Innovation in Biological, Agro-Food and Forest Systems, University of Tuscia, Via San Camillo de Lellis s.n.c., 01100 Viterbo, Italy; swati.tamantini@unitus.it (S.T.);; 2 Department of Wood Science and Technology, Biotechnical Faculty, University of Ljubljana, Rožna Dolina Cesta VIII 34, 1000 Ljubljana, Slovenia

**Keywords:** wood durability, wood decay, beech, spruce, coating, blue stain, *Gloephyllum trabeum*, *Trametes versicolor*, contact angle, adhesion

## Abstract

Lignin-based polyurethanes represent a promising strategy for developing more sustainable wood coatings by partially replacing fossil-derived polyols with renewable aromatic biopolymers. In this study, a polyurethane formulated with organosolv lignin (LPU) was synthesized and applied on two non-durable European wood species, *Fagus sylvatica* L. and *Picea abies* L., and compared with a commercial fossil-based polyurethane (CPU). Coated samples were evaluated for color stability, gloss evolution, wettability, adhesion, impact and scratch resistance, and biological durability. Accelerated ageing was performed under xenon-light irradiation, while decay resistance was assessed against *Gloeophyllum trabeum* and *Trametes versicolor*. Additional tests examined susceptibility to blue-stain fungi and surface morphology via SEM. LPU produced a matte film with intrinsically darker coloration but excellent chromatic stability and minimal gloss variation during ageing. Its initial hydrophobicity was higher on beech and comparable to CPU on spruce. Although CPU exhibited superior adhesion and slightly better mechanical resistance, LPU provided enhanced protection against blue-stain fungi—particularly on spruce—and a more uniform response to decay fungi across wood species. Overall, despite its darker appearance, the lignin-based formulation offered functional protection comparable to the commercial coating, confirming its potential as a viable bio-based alternative for above-ground wood applications.

## 1. Introduction

Wood has served as a primary construction material for millennia, valued for its mechanical performance, renewability, and ability to act as a long-term carbon sink [1,2]. In recent decades, environmental policies and the development of engineered wood products, such as cross-laminated timber (CLT), have strengthened its role in sustainable construction across Europe and beyond [3,4,5,6]. However, when used in outdoor environments, wood is exposed to abiotic and biotic agents that can severely impair its structural and aesthetic properties. Ultraviolet (UV) radiation, especially in the presence of moisture, initiates lignin photodegradation, producing chromophoric quinones responsible for greying, while blue stain and sapstain fungi can contribute to surface discoloration [7]. More critically, brown- and white-rot fungi cause extensive depolymerization of cell-wall components, leading to strength losses and dimensional instability [8,9,10].

Most native European wood species are classified as non-durable and therefore require protection for outdoor use [11]. Traditionally, wood protection has relied on biocidal preservatives; however, increasing environmental and health concerns, along with restrictions established under EU regulatory frameworks [12,13], have accelerated the search for sustainable, bio-based alternatives. These include natural compounds, nanostructured lignocellulosic materials, and chemical or physical modification processes [2,14,15,16,17,18,19]. Within coating technologies, polyurethane (PU) systems remain a benchmark because of their mechanical robustness, chemical stability, hydrophobicity, and UV durability, and several studies have confirmed their superior weathering resistance on both modified and unmodified wood substrates [19,20,21].

Despite their performance, conventional PUs are largely dependent on fossil-derived polyols and isocyanates, contributing significantly to greenhouse gas emissions. This has driven the development of bio-based polyurethane systems aligned with the principles of green chemistry and the circular economy. Among the renewable alternatives, lignin—one of the most abundant aromatic biopolymers and a major by-product of the pulp and paper industry—has gained considerable interest. Owing to its polyphenolic structure and abundance of hydroxyl groups, lignin can be directly incorporated or chemically converted into polyols suitable for PU synthesis [22,23,24]. Recent research has shown that lignin-based PUs may exhibit improved UV absorption, antioxidant activity, thermal stability, and, in some cases, antimicrobial or antifungal properties, depending on lignin source and processing [25,26]. Efforts to scale up lignin-based polyol production demonstrate increasing technological readiness for coating applications [20,27].

A previous study by Bergamasco, et al. [28] demonstrated the feasibility of synthesizing an organosolv lignin-based PU coating through reaction of lignin with isocyanate. Although the resulting bio-coating provided promising water-repellency, increased lignin content was associated with brittleness. Pure lignin coatings also exhibit intrinsic fragility and dark coloration, limiting their applicability [28,29]. Importantly, the PU formulation developed by Bergamasco, et al. [28] has not yet been evaluated on real wood substrates, and little is known about its mechanical, physicochemical, or biological performance in service-like conditions.

Recent literature further highlights the need for systematic evaluation of lignin-based coatings on actual wood [19]. Reviews and experimental studies emphasize the potential of lignin-derived polymers for bio-coatings but also point out substantial gaps in understanding their interfacial behaviour, long-term weathering resistance, and capacity to inhibit fungal colonization when applied to diverse wood species [30,31,32]. However, most available works investigate coatings on inert substrates or free-standing films; direct comparison between lignin-based and fossil-based PU coatings on wood remains limited [30].

From a circular-economy perspective, assessing whether lignin-based PU coatings can enhance the durability of non-durable wood species is therefore of considerable interest. In this study, an organosolv lignin-based polyurethane coating was applied to European beech (*Fagus sylvatica* L.) and Norway spruce (*Picea abies* (L.) Karst.), two widely used but non-durable species in Central Europe. The coatings were evaluated for resistance to abiotic degradation (UV exposure and artificial weathering), biotic agents (brown-rot fungus *Gloeophyllum trabeum*, white-rot fungus *Trametes versicolor*, and blue stain fungi), and for mechanical and surface properties including adhesion, scratch and impact resistance, wettability, and ageing behavior.

By integrating lignin into PU formulations, this work aims not only to reduce reliance on fossil-based components but also to explore whether bio-based coatings can deliver functional benefits such as improved UV protection and biological resistance. Although lignin-based PUs have been investigated for foams and solvent-borne systems, relatively few studies have examined them as coatings on real wood substrates or related lignin chemistry to performance metrics such as wettability, interfacial adhesion, or antifungal activity. This study therefore contributes to filling this gap by (I) applying an organosolv lignin-based PU to both hardwood and softwood species, (II) assessing a comprehensive set of physicochemical and durability properties, and (III) relating the observed performance to the intrinsic characteristics of lignin and the wood substrate. The results are intended to support the development of sustainable PU coatings suitable for applications corresponding to Use Class 3 (above-ground, EN 335:2013 [33]).

## 2. Results and Discussions

### 2.1. Coating Uptake and Viscosity

A clear distinction emerged between the two coating formulations in terms of uptake and viscosity. The LPU (lignin-based polyurethane coating) showed a significantly lower coating uptake compared to the CPU (commercial polyurethane coating) for both beech (4.31 ± 1.72% vs. 11.86 ± 2.34%) and spruce (6.45 ± 0.41% vs. 19.99 ± 2.47%) (Table 1). Likewise, the viscosity of LPU was markedly lower (1′50″) than that of CPU (11′22″) (Table 1). Although lower viscosity typically promotes better penetration in porous substrates, the opposite trend was observed here.

**Figure 1:**
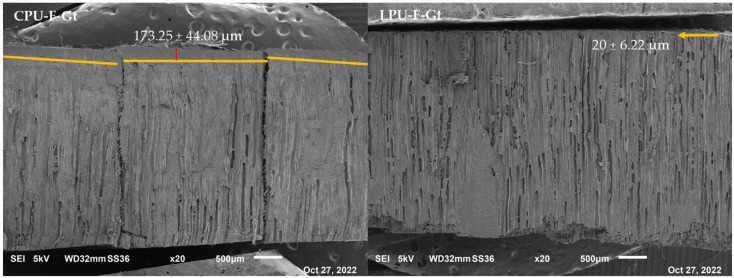
Comparison between the penetration of the prepared coatings: CPU (**left**) and LPU (**right**). As an example, coated beech wood inoculated with *G. trabeum* was selected. On the left, the yellow lines highlight the interface wood-CPU and the red line indicates the CPU thickness. On the right, the yellow arrow indicates the LPU coating.

Figure 1 shows the higher penetration of the CPU compared to the LPU, as observed by SEM. This factor is likely due to the coating application method, which involved dipping, meaning it did not allow the LPU to remain on the substrate for an extended period. However, due to its low viscosity, it could have dropped easily.

However, the result can be compared with the discussion reported in Bergamasco, et al. [28] who demonstrated that while lignin-rich polyurethanes exhibit increased stiffness and higher crosslinking density, they may also show reduced molecular mobility and substrate affinity, particularly when using organosolv lignin as a polyol precursor [28]. Moreover, the limited availability and lower reactivity of phenolic and aliphatic –OH groups in lignin, compared to conventional polyols, can lead to reduced reactivity toward isocyanates, thereby affecting coating homogeneity and the substrate’s ability to be wetted [34].

These chemical characters may further limit the penetration of LPU into the porous wood substrate, despite its lower viscosity. On Figure 1, lignin forms a very thin film that penetrates less (SEM), while the viscous CPU penetrates more (SEM). For this reason, it results in lower adhesion.

### 2.2. Artificial Weathering and Blue Stain Fungi

**Figure 2:**
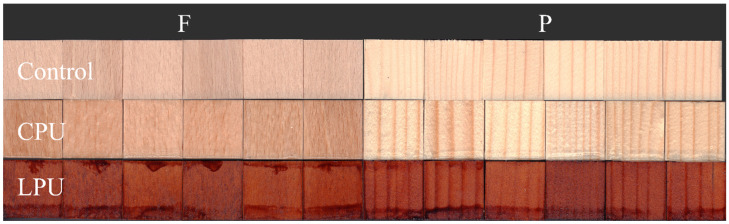
General appearance of the examined coatings (CPU and LPU) referred to uncoated wood (Control), both applied on beech (F) and spruce (P) wood.

In Figure 2, the final appearance of the treated wood before fungal exposure is reported and compared with that of the uncoated wood.

A pronounced difference in color change (∆E*) was observed between the CPU the LPU coatings applied to beech and spruce (∆E* BC-AC). CPU treatments led to moderate ∆E* values (Table 2), indicating limited visual alteration of the wood surface after coating. In contrast, LPU caused significantly higher colour shifts, reflecting the strong chromophoric contribution of lignin. This behavior is consistent with previous findings, where the presence of UV-sensitive aromatic structures in lignin was reported as a drawback for coating applications, often leading to initial darkening or yellowing [28,29]. Indeed, Bergamasco, et al. [28] and Khorshidi, et al. [35], similarly observed pronounced coloration in lignin-based polyurethane films both after application and during early exposure. During artificial weathering, lignin chromophores undergo well-known photochemical transformations, including β–O–4 cleavage, formation of phenoxyl radicals, and subsequent oxidation to o- and p-quinones, which explain the initial ΔE* increase [36,37]. However, once embedded in the polyurethane network, these chromophores exhibit reduced mobility and leaching, which can limit further color drift [21,35]. The wood substrate also plays an important role: Torcătoru and Timar [38] and Bergamasco, et al. [28] demonstrated that wood species differ in their susceptibility to light-induced discoloration, with lighter woods such as spruce showing more visible changes when coated with pigmented or chemically active finishes.

The comparatively low ∆E* values observed for CPU (BC-AC) align with the typical behavior of fossil-based polyurethane coatings, which are generally formulated to maintain wood’s natural appearance [2]. Pavlič et al. [3] showed that only chemical modification or bleaching can help mitigate the effect of browning. The dark visual impact may limit the application in contexts where the preservation of the natural wood color is essential.

Color stability during ageing (120 h and 240 h) confirmed these trends. CPU showed a progressive increase in ΔE*, indicating ongoing UV-induced yellowing. Conversely, LPU presented much smaller ΔE* variations after the initial shift, despite containing photoactive groups such as phenolic hydroxyls and carbonyls known to absorb UV radiation and undergo chromophore transformations [39,40]. The cumulative ΔE* between AC and 240 h remained low for LPU, demonstrating improved stabilization over time.

These results align with previous studies, which indicate that lignin, although sensitive to initial UV-induced oxidation, may contribute to improved oxidative stabilization when incorporated into polyurethane networks [21]. Lignin’s inherent UV-absorbing capacity and radical scavenging behavior can reduce ongoing photodegradation, thereby helping to maintain color over extended exposure periods [22,41]. In contrast, conventional aromatic polyurethane resins, such as CPU, may yellow progressively due to oxidative chain scission and chromophore formation [42]. Overall, despite higher initial ΔE* values, LPU demonstrated superior resistance to long-term color drift, which is advantageous for sustainable wood coatings exposed to outdoor or high-irradiance environments.

Gloss measurements conducted at different angles (20°, 60°, and 85°) re-veal distinct behaviors between the CPU and the LPU coatings on both beech and spruce wood substrates, with notable differences based on the orientation of measurement relative to the wood grain (P—parallel or O—orthogonal) (Table 3). CPU-treated beech samples exhibited a rapid increase in gloss during the first 120 h of artificial ageing, reaching peak values of 65.7 (60°/P), followed by a progressive decline over the next 120 h. This pattern is consistent with previous studies reporting gloss enhancement due to initial film leveling, followed by photodegradation and surface roughening [43,44]. In contrast, LPU coatings exhibited significantly lower gloss levels across all conditions, with values consistently below 12 at 60° and displaying minimal variation over time and direction. This stability is indicative of LPU’s more matte and isotropic finish, likely related to the influence of lignin-based polyols on film morphology and light scattering behavior [22,45]. The difference in gloss behavior between CPU and LPU is particularly evident when comparing the directionality of measurements. CPU showed a strong anisotropy, with gloss values in the parallel direction significantly exceeding those in the orthogonal direction, especially on beech. This is attributable to the alignment of wood fibers and surface reflection dynamics, as previously observed in coated coniferous and hardwood species [46]. On the other hand, LPU coatings exhibited a more uniform gloss response between orientations, suggesting improved substrate penetration and a more homogeneous surface topology.

Furthermore, the presence of lignin in LPU may contribute to improved UV stability and reduced gloss loss. Lignin’s intrinsic ability to absorb UV radiation and scavenge free radicals has been demonstrated to mitigate photodegradation in polymer coatings [35,47]. Khorshidi, et al. [21] reported that lignin-based polyurethanes significantly enhance photostability, maintaining gloss and surface integrity over extended exposures. These results suggest that LPU formulations could serve as a viable sustainable alternative to conventional polyurethanes in applications requiring long-term gloss retention and reduced optical degradation.

### 2.3. Contact Angle

**Figure 3:**
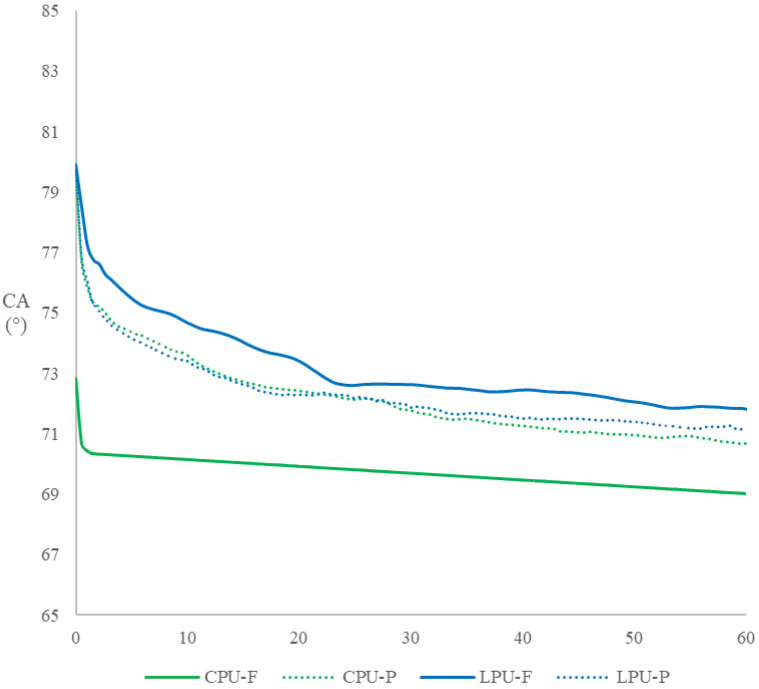
Contact angle analysis of the coatings applied on beech and spruce wood. CPU: Commercial Polyurethane. LPU: lignin-based polyurethane. F: *F. sylvatica*. P: *P. abies*.

Figure 3 presents the trend of the dynamic contact angle (CA) (1 min) for the coatings studied in the present work.

The contact angle (CA) measurements over 60 min revealed significant differences in the wetting behavior of the commercial polyurethane (CPU) and lignin-based polyurethane (LPU) coatings on *F. sylvatica* (F) and *P. abies* (P). In the beech wood, there is a notable difference in bsehavior between the CPU and LPU, as LPU (80) exhibits a significantly higher contact angle from the beginning, compared to CPU (80° vs. 73°). In both cases, the contact angle decreases over time, and after 60 s the difference, although still remarkable, becomes less evident (73° for LPU vs. 70° for the commercial CPU with a difference of 3°). The gradual reduction in contact angle suggests a moderate increase in surface wettability, possibly due to surface rearrangement or absorption phenomena [34,35].

There is only one character that is not totally favorable in LPU, which is a small fluctuation in the contact angle in LPU, indicating a non-homogeneous surface.

In spruce, the difference between CPU and LPU is considerably smaller; in fact, the difference in contact angle is barely noticeable even in the initial stages (for both the contact angle is 80°). As time increases, the contact angle decreases, though less markedly than observed in beech, and up to the end of the test the difference in wettability between LPU and CPU remains negligible.

The differences between the two substrates for each coating type are likely related to their distinct anatomical and chemical surface characteristics, which influence coating adhesion and surface energy [48]. The higher initial CA values observed for LPU coatings can be attributed to the hydrophobic nature of lignin and its impact on surface chemistry, consistent with previous studies that demonstrate lignin’s influence on coating hydrophobicity [49,50]. These findings highlight that while LPU coatings present initially more hydrophobic surfaces, their wettability evolves over time, a factor to consider for applications requiring controlled moisture interactions.

It cannot be ruled out that the high heterogeneity in the anatomy of beech wood influenced the result, due to a greater presence at that point of a fibrous structure rather than vessels, a factor that may have contributed to the higher contact angle value.

### 2.4. Impact Test

Results of the impact test are reported in Table 4.

The impact resistance of the coated wood surfaces was assessed by evaluating the level of damage following controlled drop tests at two heights (25 cm and 100 cm). Both coatings exhibited comparable performance on beech wood, with damage scores of 4 at both heights for both the CPU and LPU. On spruce wood, CPU coatings showed slightly lower damage scores (3 at both heights), indicating marginally better resistance to localized mechanical stress compared to beech. Conversely, LPU coatings exhibited a uniform damage score of 4 across both species and heights, suggesting a consistent but slightly higher susceptibility to impact-induced deformation.

These results suggest that the LPU coating may have a slightly lower mechanical damping capacity under high-strain conditions compared to CPU, possibly due to differences in crosslinking density or elastic modulus. Nevertheless, the performance of LPU remains within an acceptable range, demonstrating that the incorporation of lignin does not significantly compromise impact resistance. Previous studies have shown that lignin-based polyurethanes can present lower flexibility or toughness depending on lignin type and content, which can influence impact behavior [52,53].

### 2.5. Scratch Test

The results of the scratch test are reported in Table 5.

The scratch resistance of the coatings was evaluated by measuring the maximum indentation width under increasing loads (5 N and 10 N). On *F. sylvatica*, the LPU coating exhibited slightly superior resistance, with narrower indent widths (0.3 mm at 5 N and 0.4 mm at 10 N) compared to the CPU coating (0.4 mm and 0.6 mm, respectively). This suggests a greater surface hardness or better load distribution in the LPU formulation under mild to moderate force.

On *P. abies*, LPU again outperformed CPU at 5 N (0.4 mm vs. 0.7 mm), both coatings exhibited an equal maximum indent width of 1.2 mm at 10 N, indicating that under higher stress, CPU and LPU behave in the same way under indentation. At higher loads, the indentation is likely predominantly influenced by the wood’s structure, and since spruce is softer than beech, the resulting final value is higher.

This outcome might be compared to the results obtained with previous studies that demonstrate how the interaction between coating rigidity and wood substrate anisotropy influences scratch propagation and plastic deformation [49,55]. The improved performance of LPU at lower loads may be attributed to the aromatic nature of lignin, which can increase crosslink density and microhardness when properly incorporated into polyurethane networks [56,57]. Interestingly LPU has less indentation compared to the CPU, except for the spruce under high load.

### 2.6. Adhesion Test

The results of the adhesion test are reported in Table 6.

The CPU demonstrated higher adhesion stress values compared to the LPU on both wood species. Specifically on beech, CPU showed an average adhesion stress of 7.80 ± 0.46 MPa, whereas LPU exhibited a slightly lower value of 7.17 ± 0.38 MPa. This difference, although moderate, suggests a comparable adhesive performance on hardwood substrates. On spruce, the difference was a bit more pronounced, with CPU achieving 4.77 ± 0.11 MPa compared to 3.58 ± 0.30 MPa for LPU, indicating reduced adhesion of the lignin-based formulation on softwood. This behavior may be related to spruce anatomy and its higher resin content, which influence coating–substrate interactions [48,59]. Further, spruce tends to be less permeable than beech, due to its more compact tracheid structure and the presence of resinous deposits that obstruct fluid uptake. These characteristics limit coating penetration and reduce the effective interfacial area available for mechanical interlocking. Moreover, the chemical composition of spruce lignin—predominantly guaiacyl units—provides fewer accessible phenolic and aliphatic hydroxyl groups compared to the more syringyl-rich lignin of hardwoods. The lower proportion of reactive OH functionalities at the wood surface may further hinder interfacial bonding mechanisms, particularly for LPU, whose adhesion relies in part on interactions between lignin-derived phenolic hydroxyls and wood hydroxyl groups [48,59]. The reduced adhesion of LPU can also be associated with intrinsic factors of the coating formulation, including the lower overall reactivity of lignin phenolic hydroxyls with the wood substrate and the higher crosslinking density of the polymer network, which may restrict chain mobility and wetting. Additionally, the denser lignin-rich matrix may impede mechanical anchoring within the porous microstructure of softwoods, thereby contributing to the lower adhesion stresses observed in spruce [34,48]. Despite this, the adhesion values of LPU remain within acceptable limits for protective coatings, demonstrating that lignin incorporation does not critically compromise interfacial bonding [49]. Previous studies have highlighted the potential of lignin-based polyurethanes to form strong chemical interactions with wood surfaces, although the mechanical adhesion may vary depending on the lignin source and formulation parameters [60,61].

### 2.7. Artificial Weathering

**Figure 4:**
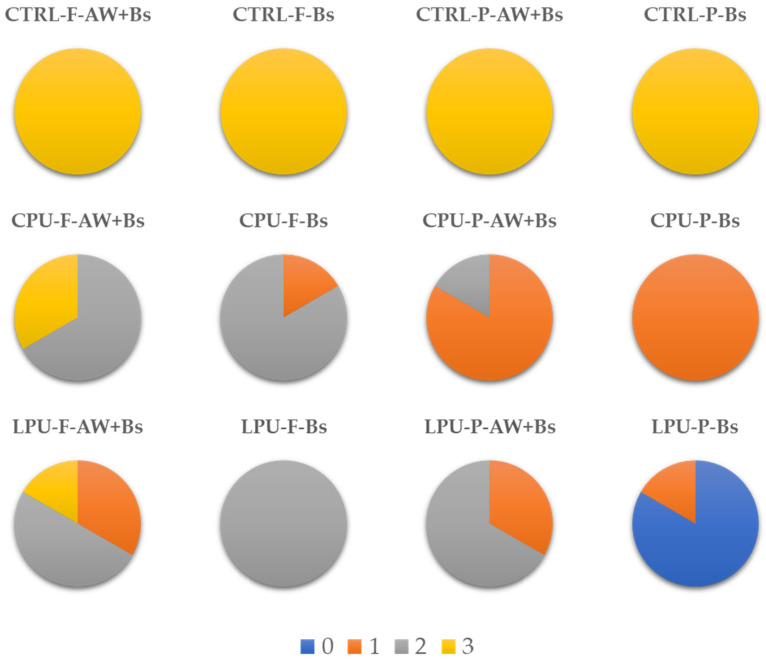
Artificial weathering (AW) and blue stain (Bs) results. Control sample (CTRL); Beech (F) and Spruce (P) wood sample coated with commercial polyurethane (CPU) and lignin-based polyurethane (LPU).

The accelerated aging and blue stain inoculation tests revealed clear distinctions in the protective efficiency of CPU and LPU coatings on beech (F) and spruce (P) wood substrates (Figure 4). Uncoated samples (CTRL) uniformly exhibited class 3 ratings—irrespective of artificial weathering (AW)—indicating extensive blue stain fungal penetration and reaffirming the vulnerability of untreated wood (Figure 4).

CPU-coated samples displayed intermediate performance, with beech (CPU-F) surfaces largely sustaining class 2 colonization, particularly under AW + Bs, whereas Bs alone led to slightly lower fungal presence. Conversely, spruce (CPU-P) samples experienced more significant degradation, predominantly rated as class 1 (EN 152:2011 [62]) in both AW + Bs and Bs conditions, suggesting a reduced efficacy of CPU on spruce.

LPU coatings exhibited the most favorable outcomes. On beech (LPU-F), the predominant Class 2 rating shifted toward Class 1 under Bs, indicating moderate improvement over CPU. Notably, spruce (LPU-P) samples were often in class 0 and 1, with class 0 dominating under Bs exposure. This suggests a potent antifungal effect of LPU, particularly on spruce wood. These results align with recent evidence indicating that lignin-based coatings exhibit superior UV-blocking, radical-scavenging, and antimicrobial properties [35,63,64,65]. Hence, LPU presents an environmentally promising alternative to CPU, significantly enhancing resistance to fungal colonization, especially on *P. abies*. Coating degradation is mainly driven by UV-induced aromatic oxidation, hydrolysis of urethane bonds, and enzymatic softening of the polymer by fungi [42,66]. Strategies to mitigate degradation include, use of UV absorbers, or incorporation of nanofillers such as CNCs [22].

These findings highlight that the lignin-based polyurethane coating offers competitive or superior protection compared to commercial formulations in respect to the blue stain risk, especially on spruce substrates, and may benefit further from its potential environmental advantages.

### 2.8. Decay Test

**Figure 5:**
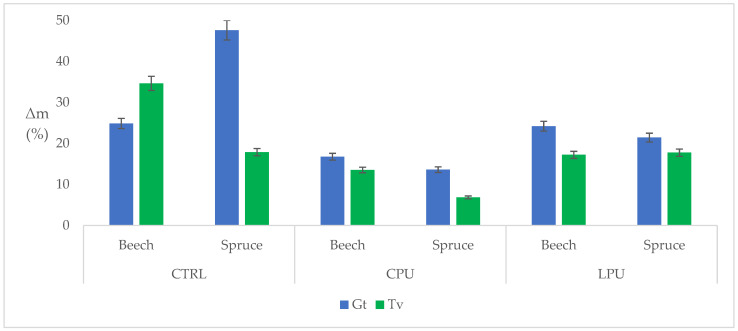
Mass loss (Δ_m_) percentage after the decay test of brown wood rot (Gt) and white wood rot (Tv). CTRL: Control sample of Beech and Spruce wood. CPU: Commercial polyurethane coating. LPU: Lignin-based polyurethane.

Figure 5 presents the mass loss (Δₘ) of beech and spruce samples exposed to *Gloeophyllum trabeum* (Gt, brown-rot) and *Trametes versicolor* (Tv, white-rot). As expected, the uncoated control (CTRL) samples exhibited the highest degradation, meaning that the fungal strains were vital, and the wood was susceptible to fungal decay (as reported in EN 350:2016 [67]), in agreement with previous literature [68,69].

A fungus-specific trend can be observed: Gt was more aggressive on spruce (Δ_m_ = 47.63% than on beech (24.87%), whereas Tv caused greater degradation on beech (34.65%) than spruce (17.88%). This behavior is consistent with fungal ecology, where brown-rot species preferentially degrade softwoods, while white-rot fungi degrade hardwoods more efficiently due to their ligninolytic enzymatic systems. This contrasting decay pattern arises from fundamental anatomical and chemical differences between softwoods and hardwoods, as well as the distinct enzymatic systems of brown- and white-rot fungi. Brown-rot fungi rely primarily on a combination of non-enzymatic Fenton chemistry and selective carbohydrate-degrading enzymes to rapidly depolymerize cellulose and hemicellulose, while largely modifying—but not extensively mineralizing—lignin. Softwoods contain simpler lignin structures (mostly guaiacyl units) and more uniform tracheid-based anatomy, which allows brown-rot fungi to more easily diffuse low-molecular oxidative agents and access the polysaccharide-rich cell wall. In contrast, hardwoods possess more complex lignin (higher syringyl content) and heterogeneous anatomy, which slows the diffusion of reactive oxygen species and reduces the efficiency of the brown-rot decay mechanism. Conversely, white-rot fungi produce a full suite of ligninolytic enzymes (laccases, peroxidases, MnP, LiP, VP) capable of degrading the diverse lignin structures characteristic of hardwoods. The greater abundance of syringyl-rich lignin in hardwoods is more susceptible to these oxidative enzymes, enabling more extensive delignification and overall mass loss. As a result, white-rot species such as *Trametes versicolor* typically cause markedly higher degradation in hardwoods than in softwoods, fully aligning with the ecological pattern observed in respective results [66,70,71].

Coated samples demonstrated improved resistance. On average, coatings reduced mass loss to 15.59% (Gt) and 13.84% (Tv), indicating a protective effect of both commercial polyurethane (CPU) and lignin-based polyurethane (LPU). While the reduction was not complete, it confirms that surface coatings inhibited fungal colonization and activity to some extent, as also reported by Humar and Lesar [68]. Among the two coatings, CPU exhibited greater efficacy overall. For instance, CPU-treated spruce showed a significant decrease in Δₘ to approximately 13.61% (Gt) and 6.85% (Tv), while beech reached 16.76% and 13.54%, respectively. These values reflect moderate fungal resistance, with a better performance against white-rot fungus and by spruce wood.

Although LPU is less effective overall, it indicates a noticeable decrease in mass loss, which tends to homogenize the degradation pattern between brown and white rot. Moreover, it reduces the influence of the wood substrate, leading to a more uniform response across species. Ultimately, both fungi behave similarly on LPU-treated samples. Nevertheless, spruce still shows the best performance against Gt, and slightly better results than beech when exposed to Tv. Nonetheless, LPU seems to act selectively, reducing the damage caused by white rot fungi (TV) in beech, and it appears to be even more effective against brown rot fungi in conifers.

The improved resistance of LPU to *T. versicolor* in beech can be attributed to a combination of biochemical inhibition and structural reinforcement arising from the incorporation of lignin into the polyurethane network. Lignin introduces a high density of phenolic and methoxylated aromatic units, which interfere with the oxidative enzyme systems characteristic of white-rot fungi—most notably laccases and class II peroxidases. These enzymes are central to the lignin-depolymerization cascade, and their suppression directly limits the fungus’s ability to initiate the early stages of cell-wall erosion in hardwoods [65,72]. In parallel, lignin-derived phenolics function as powerful radical scavengers, neutralizing reactive oxygen species and intermediate radicals generated during fungal metabolism. By attenuating these redox-driven reactions, the LPU matrix effectively disrupts the oxidative cycles required for sustained enzymatic activity, thereby slowing hyphal advancement and reducing the metabolic efficiency of *T. versicolor*.

Beyond biochemical interactions, the presence of lignin leads to the formation of a denser, more rigid, and structurally cohesive polyurethane matrix, which decreases internal free volume and restricts mass transport processes. This reduction in free volume lowers water uptake, thereby limiting moisture availability at the coating–wood interface—an essential factor for fungal germination, enzyme secretion, and diffusion. The improved dimensional stability of the LPU coating also minimizes microcrack formation, reducing potential entry points for fungal hyphae. Moreover, the increased rigidity of the lignin-enriched polymer poses a mechanical obstacle to hyphal penetration, adding an extra defensive layer against colonization. Together, these intertwined biochemical and physical mechanisms provide a plausible explanation for the selective antifungal performance of LPU, particularly its enhanced capacity to inhibit white-rot fungi such as *T. versicolor* on hardwood substrates [22,39,63,66].

## 3. Materials and Methods

### 3.1. Materials

In this study, two of the most common European species were selected: beech wood (*Fagus sylvatica* L.) and Norway spruce wood (*Picea abies* (L.) H. Karst.). They were selected because they are common in the wood market and because of their low durability, which makes the two species very interesting to test the improved durability due to coatings. In fact, in order to study the coatings, no anti-fungal effect should come from the wood itself. These wood species exhibit distinct intrinsic properties that are expected to influence the coating performance across the different tests conducted. Beech contains higher syringyl/guaiacyl lignin ratio, greater porosity, and higher density, which influence coating penetration and adhesion. Spruce, characterized by lower density and high extractive content, shows different wetting and fungal susceptibility. Wood samples were taken from one board, cut in tangential direction, and conditioned in the lab at 65% relative humidity (RH) and 20 °C for one year. Samples for durability test against have dimensions of 30 × 15 × 9 mm^3^ (L × T × R), as an adaptation of the standard EN 113:2020 [73]. Samples for blue stain, xenon exposure and contact angle have dimensions of 25 × 25 × 5 mm^3^, as the surface is important in this kind of tests. Samples for physical tests (impact, scratch, and adhesion tests) have dimensions of 150 × 70 × 10 mm^3^. Although samples were smaller than standard dimensions, all treatments were tested under identical conditions, enabling valid comparative analysis. Previous studies have shown that relative differences in coating performance remain reliable even with scaled-down samples [28,74,75].

The fossil-based polyurethane (CPU) was provided by the company Finedin S.r.l. (Taviano, Italy). Main functions of this transparent coating are the mechanical protection of wood against impacts and scratches in indoor and outdoor conditions.

For the synthesis of the lignin-based polyurethane (LPU), hardwood (beech) Organosolv Lignin (OSL) was supplied by Fraunhofer Center for Chemical-Biotechnological Process. Desmodur^®^L75 is an aromatic polyisocyanate based on toluene diisocyanate (TDI), it was supplied by Covestro LLC (Leverkusen, Germany). Thetrahydro-2-methylfuran (2-MeTHF) was obtained from Carlo Erba Reagents (Cornaredo, Italy). The chemical structure of the two polyurethane systems differs substantially. CPU is based on conventional polyether–polyol and aliphatic isocyanate chemistry, resulting in a flexible polymer network with high chain mobility and uniform urethane linkages. In contrast, LPU incorporates organosolv lignin as a polyol substitute. Lignin provides aromatic rings, phenolic hydroxyl groups, and sterically hindered aliphatic OH groups, which participate in urethane formation but also introduce rigidity into the polymer backbone. The presence of condensed aromatic structures increases crosslinking density and reduces polymer chain mobility, producing a more compact and stiffer matrix.

The nutrient medium for decay test was potato dextrose agar (PDA) (Difco^TM^, Sparks, MD, USA).

### 3.2. Methods

#### 3.2.1. Coatings Preparation and Application

The biobased PU synthesis was obtained using a modified protocol of Bergamasco, et al. [28]. The lignin was dispersed in 45 mL of 2-MeTHF with a dispersion degree equal to 60% (wt:vol) and stirred magnetically at 400 rpm for 30 min at room temperature (25 ± 2 °C). After that, Desmodur^®^L75 was added to the lignin to reach the ratio 3:1 (*w*:*w*) isocyanate:lignin ratio. For a complete dissolution of isocyanate and eventual polyurethane formation, the solutions were left under magnetic stirring (at 400 rpm) for additional 60 min at room temperature (25 ± 2 °C). Finally, wood samples were dipped in the PU formulations (CPU and LPU) once, left for 5 s and let dry for 48 h. No inert gas atmosphere was required, as previous tests confirmed negligible oxidation in these conditions. The resulting prepolymer solution was immediately used for coating preparation to minimize viscosity changes. All reactions were performed in a fume hood to ensure constant temperature and humidity control.

Six samples of 30 × 15 × 9 mm^3^ (48 total samples) and six samples of 25 × 25 × 5 mm^3^ (48 total samples) per each treatment were tested (96 total samples).

#### 3.2.2. Coating Uptake

The Samples mass was measured using an electronic analytical scale (EP 320A, LOTRIČ Metrology Group, Selca, Slovenia). Samples were weighed before coating application and after a 48-h drying period.

#### 3.2.3. Optical Contact Angle

Optical contact angle (CA) measurements on the formulations were performed at room temperature using the optical tensiometer Attension Theta Flow (Biolin Scientific Group, Gothenburg, Sweden). Six samples per treatment were tested. A drop of 4 μL of distilled water was released on the coated samples and time of observation was 60 s per specimen.

Data elaborations were conducted using the software OneAttension v4.1.2 (r9576), provided by Biolin Scientific Group (Gothenburg, Sweden).

#### 3.2.4. Impact Test

The resistance to impact was determined following the standard ISO 4211-4:1988 [51]. A steel cylinder of 500 ± 5 g was placed at different heights (25 and 100 cm) for free fall onto a steel ball (Ø = 14 mm) placed on the surface of the coating. After impact, the surface was examined with a magnifier (10×) and the impact resistance of the coating was evaluated using numerical grades according to the standard. Three replicates were performed for each species and drop height.

#### 3.2.5. Scratch Test

The scratch test was carried out following the standard EN ISO 1518-1:2019 [54]. A spring test pencil (Model 318, Erichsen GmbH & Co. KG, Hemer, Germany) was used for the purpose. A needle is inserted with a tip that is half a sphere, 1 mm in diameter. The measurements of the indent were recorded at a load of 5 and 10 N. The scratch length was at least 60 mm and the scratch speed was 30–40 mm/s. The scratch was performed perpendicular to the grain direction. The widest indent width was recorded for each species and load.

#### 3.2.6. Adhesion Test

Pull-off adhesion test was carried out according to the standard EN ISO 4624-2016 [58]. Aluminum dollies (Ø = 20 mm) were glued on the surface of the coatings using a 2-component polyurethane adhesive. After a curation period of 24 h, the coating around the dollies was carefully cleaned down to the substrate in order to isolate the glued zone from the rest of the coating layer. The tensile stress applied to peel off the coating from wood surface was measured by using a Defelsko Positest^®^ Adhesion tester (Defelsko instruments corporation, Ogdensburg, NY, USA).

#### 3.2.7. Artificial Weathering and Blue Stain Fungi

The artificial weathering (AW) process of the samples (25 × 25 × 5 mm) was carried out using the Atlas SUNTEST XXL + chamber (Atlas Material Testing Technology, Mount Prospect, IL, USA), with the daylight glass filter on. Conditions of the chamber were an irradiance of 0.35 W/m^2^, an irradiance wavelength of 340 nm, the temperature of the chamber was 38 °C, the RH was 55%, as stated in the standard ISO 16474-2:2013 [76]. The uncoated (CTRL) and coated samples (with CPU and LPU) were exposed for 240 h and color and gloss values were recorded before and after coating application and after 120 h and 240 h of xenon exposure.

The color difference was calculated using the following equation (Equation (1)):

(1) $${∆E}^{*}=\sqrt{{\left(L_{2}^{*}-L_{1}^{*}\right)}^{2}+{\left(a_{2}^{*}-a_{1}^{*}\right)}^{2}+{\left(b_{2}^{*}-b_{1}^{*}\right)}^{2}}$$

where: L* is the lightness (0 to 100); a* is the coordinate for red/green values (−120 to 120); b* is the coordinate for blue/yellow values (−120 to 120); Index 1 stands for the reference sample; Index 2 stands for the sample to analyze.

EN 152:2011 [62] standard was followed for the blue stain procedure. The specimens exposed to xenon lamps and the specimens not exposed (25 × 25 × 5 mm^3^) were tested for the blue stain fungi (96 total samples), here after abbreviated with AW + Bs and Bs, respectively. The test organisms were *Aureobasidium pullulans* (de Bary) G. Arnaud and *Sclerophoma pithyophila* (Corda) Höhn.

#### 3.2.8. Decay Test

The decay test (D) was performed according to the standards EN 113-1:2021 and EN 113-2:2021 [73,77].

Before starting, the weight of the coated samples was recorded, then they were placed in the oven for 48 h at 60 ± 2 °C in order to not damage the coatings. The weight of the specimens was also recorded after the samples were removed from the oven.

PDA was prepared and poured into sterilized Petri dishes (Ø = 85 mm, h = 15 mm). The samples and the filled Petri dishes were then sterilized in an autoclave at a temperature of 121 °C and pressure of 150 kPa for 20 min. From this point on, everything was done in a sterile environment using sterile equipment. Plastic meshes (HDPE) were placed over the PDA in the Petri dishes in order to prevent direct contact between the samples and the growth medium. Then the Petri dishes were inoculated with a white rot fungus (*Trametes versicolor* (L.: Fr.) Pilàt) and a brown rot fungus (*Gloeophyllum trabeum* (Pers.) Murrill). The fungal collection of the Biotechnical Faculty, University of Ljubljana, Slovenia, provided the fungal cultures Raspor, et al. [78]. A total of 48 samples of 30 × 15 × 9 mm^3^ (six samples per treatment) in 16 Petri dishes (3 samples per Petri dish) were exposed in incubation chamber (T = 25 °C; RH = 85%) for 12 weeks.

After this period, mycelium was carefully removed from the surface of the samples, then the samples were weighed for quality control and oven dried at 60 ± 2 °C for 48 h. The samples were then weighed, and the samples mass variation (Δm) was calculated according to the following equation (Equation (2)) by Akitsu, et al. [79].

(2) $$∆m\left[\%\right]=\frac{m_{0}\left[g\right]-m_{1}[g]}{m_{0}[g]}100$$

where m_0_ is the oven dry mass of the sample prior to fungal exposure and m_1_ is the oven dry mass of the sample after the inoculation and incubation of 12 weeks [units of measurement are in square brackets].

#### 3.2.9. SEM

For Scanning Electron Microscopy (SEM), samples were attached to aluminium stubs using carbon tape and sputter-coated with gold in a Balzers MED 010 unit. The observations were made by a JEOL JSM 6010LA electron microscope (Akishima, Tokyo, Japan) using Secondary Electrons (SE). The analysis was carried out on one representative decay test sample per coating in order to investigate the level of damage.

#### 3.2.10. Statistical Analysis

Data storing and elaboration was performed by means of Excel (Microsoft 365, Microsoft, Redmond, WA, USA). The statistical analyses were carried out using Minitab (v18.1, Minitab Inc., State College, PA, USA) and Python (v3.12, Python Software Foundation, Beaverton, OR, USA). Test of normality (Anderson-Darling methos) was done in order to identify which hypothesis test fitted the data. In case of normality distribution, the ANOVA test was chosen, and a Tukey test performed for the statistical significance, otherwise, for non-parametrical data a *t*-test was carried out.

#### 3.2.11. Experimental Design

**Figure 6:**
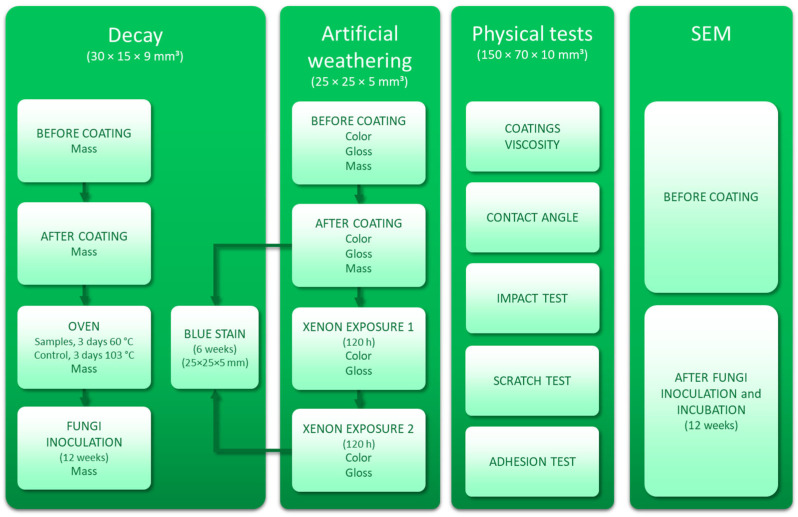
Overview of the experimental workflow, including decay assessment, artificial weathering (color, gloss, and mass measurements before and after coating, Xenon exposure, and blue stain test), physical tests (viscosity, contact angle, impact, scratch, and adhesion), and SEM analyses performed before coating and after fungal incubation.

The experimental design is synthetized in Figure 6.

## 4. Conclusions

The results show that the lignin-based polyurethane (LPU) represents a credible and sustainable alternative to conventional polyurethane coatings. Although presenting a darker coloration due to the lignin component, the resulting film is stable and compact, maintaining a nearly unchanged matte finish over time. Its physical performance varies according to the wood species: on beech, LPU exhibits higher water repellency than the commercial formulation (CPU), while on spruce the differences between the two coatings are minimal. Adhesion and mechanical resistance of LPU, although slightly lower, remain adequate for above-ground applications.

From a biological perspective, LPU provides protection comparable to conventional products. The coating shows improved resistance to blue-stain fungal colonization—particularly on conifers—and a more uniform response to decay fungi, reducing the influence of the intrinsic characteristics of different wood species. The protection against fungal degradation is especially effective against brown rot in conifers and, to a lesser extent, white rot in beech, likely due to the biological activity of lignin’s phenolic groups.

In summary, LPU combines satisfactory functional performance with a reduced environmental impact, confirming the potential of lignin-based polyurethanes for use class 3 applications. Further developments, such as chemical modification of lignin or the use of compatibilizers, could enhance adhesion and mechanical behavior, bringing these materials closer to fully bio-based solutions.

## Figures and Tables

**Table 1:** Coating uptake (%) for the treated samples and viscosity of the two formulations.

	Coating Uptake		Viscosity
	Beech	Spruce	
CPU	11.86 ± 2.34%	19.99 ± 2.47%	11′22″
LPU	4.31 ± 1.72% *^,1^	6.45 ± 0.41% *^,1^	1′50″ *^,1^

^1^ Statistical difference is reported as: * *p* < 0.001.

**Table 2:** Color variation (ΔE*) of beech and spruce wood surfaces coated with fossil-based polyurethane (CPU) and lignin-based polyurethane (LPU) after artificial weathering. ΔE* values are reported for different exposure intervals: before coating (BC), after coating (AC), after 120 and 240 h of exposure (120 h and 240 h) and as cumulative color change (AC–240 h).

	ΔE* (BC-AC)		ΔE* (AC-120)		ΔE* (120–240)		ΔE* (AC-240)	
	Beech	Spruce	Beech	Spruce	Beech	Spruce	Beech	Spruce
CPU	10.3 ± 1.8	4.9 ± 1.4	15.0 ± 3.9	20.0 ± 1.3	4.1 ± 0.8	6.1 ± 0.7	18.8 ± 3.8	25.6 ± 2.0
LPU	37.3 ± 2.0	45.2 ± 1.4	7.1 ± 0.7	8.7 ± 0.9	1.1 ± 0.5	1.5 ± 0.5	7.9 ± 0.7	9.4 ± 0.6

**Table 3:** Gloss analysis of the treated samples is reported as gloss unit (GU). BC: Before coating; AC: After coating; 120 h: After 120 h of artificial weathering (AW) exposure; 240 h: After 240 h of AW exposure. P: Light beam parallel to the wood grain; O: Light beam orthogonal to the wood grain. CPU: Commercial polyurethane coating; LPU: lignin-based polyurethane coating.

		BC						AC						120 h						240 h					
		P			O			P			O			P			O			P			O		
		20°	60°	85°	20°	60°	85°	20°	60°	85°	20°	60°	85°	20°	60°	85°	20°	60°	85°	20°	60°	85°	20°	60°	85°
CPU	Beech	1.1	4.4	8.1	1.0	3.6	2.7	22.4	65.7	45.2	19.3	54.3	43.1	12.5	56.4	35.8	11.4	43.5	34.9	22.2	52.1	34.6	10.3	40.1	34.4
	Spruce	1.3	5.2	2.9	1.3	4.4	1.5	10.9	52.3	31.8	10.7	41.5	29.9	7.6	41.4	24.5	7.0	33.0	24.0	7.6	40.3	24.0	6.6	31.6	23.8
LPU	Beech	1.0	4.4	8.5	1.0	3.6	4.7	1.8	11.4	8.8	2.0	9.1	4.6	1.9	11.1	9.6	2.0	10.2	6.2	1.8	11.0	10.1	1.8	9.0	5.0
	Spruce	1.4	5.9	4.1	1.3	4.5	1.5	1.0	7.1	5	1.0	5.6	2.4	1.1	6.8	4.8	1.3	7.4	3.0	1.0	6.6	4.1	1.1	6.0	2.4

**Table 4:** Level of damage (ISO, 4211-4:1988 [51]) for the impact test on the acrylic coatings. The sphere was dropped at two different heights (25 and 100 cm).

	Level of Damage			
Species	*Fagus*		*Picea*	
Height [cm]	25	100	25	100
CPU	4	4	3	3
LPU	4	4	4	4

**Table 5:** Scratch test (EN ISO 1518-1:2019 [54]) results.

	Maximum Indent Width [mm]			
Species	*Beech*		*Spruce*	
Load [N]	5	10	5	10
CPU	0.4	0.6	0.7	1.2
LPU	0.3	0.4	0.4	1.2

**Table 6:** Results of the adhesion test (EN ISO, 4624:2016 [58]) for the two wood species.

	Adhesion Stress [MPa]	
Species	Beech	Spruce
CPU	7.80 ± 0.46	4.77 ± 0.11
LPU	7.17 ± 0.38	3.58 ± 0.30

## Data Availability

Data available upon request.
